# Platelet-Rich Plasma (PRP) for Endometrial Treatment Efficacy and Safety in Assisted Reproductive Technology: A Comprehensive Review

**DOI:** 10.7759/cureus.59728

**Published:** 2024-05-06

**Authors:** Priti Karadbhajne, Hellen Y Dzoagbe, Akash More

**Affiliations:** 1 Clinical Embryology, Datta Meghe Institute of Higher Education and Research, Wardha, IND; 2 Pharmacy, Datta Meghe Institute of Higher Education and Research, Wardha, IND; 3 Nursing, Public Health Nurses' School, Accra, GHA

**Keywords:** in vitro fertilization, infertility, platelet and growth factor, implantation rate, endometrial receptivity

## Abstract

Platelet-rich plasma (PRP) is among the new ground-breaking methods called for endometrial disorders detected in assisted reproductive technology (ART). This research work takes account of both a systematic literature review and an exploration of the molecular connections. The report aims to present the capabilities and benefits of the PRP in ART and the nonconformance and dangers involved in its use in ART. However, all these stages of ART need conducive endometrium, live micro embryo, and coordinated interaction between the blastocyst and the uterus. Despite all ART has achieved, implantation failure still exists as a problem having one quarter being attributed to the absence of the endometrial receptivity level. The review points to a corresponding increase in the role of autologous PRP in promoting cell proliferation, neo-angiogenesis, and anti-inflammatory effects to facilitate effective endometrial receptivity. The outcome of prior trials with the PRP approach proved to be positive for women with adenomyosis, thin endometrial lining, recurring implantation failure, chronic endometritis, and Asherman’s syndrome. Challenges still exist in the proper placement of PRP for all women with infertility problems as well as how it works for individuals with blood disorders and infections. This study will look into the safest number of doses, the time of acting, and the possible future health hazards that both mother and child may face.

## Introduction and background

Platelet-rich plasma (PRP) is a remarkable therapeutic modality used in assisted reproductive technology (ART), primarily to treat fetal conditions. Strong interactions with PRP and uterine tissue have attracted considerable interest due to their ability to increase endometrial receptivity and enhance reproductive outcomes. Below, valuable insights have been gained into the efficacy and safety of PRP in endometriosis in a complex clinical situation characterized by limited treatment options [1]. Uterine thickness is an important determinant of pregnancy outcomes for infertility. The presence of a thin uterus, defined as less than 7 mm, is a significant barrier to successful pregnancy if hematopoietic stem cell transplantation is used, even if PRP is used. PRP has shown the ability to increase thinning and induce embryo implantation in individuals with a uterus in the thinning process [2,3]. Nevertheless, a comprehensive evaluation of the efficacy and safety of PRP in ART is still a matter for further research.

The therapeutic effect of PRP on the placenta is attributed to its ability to provide essential factors for tissue regeneration, angiogenesis, and cell proliferation. Research has emphasized the importance of PRP for uterine thickening, vascularity, and thickness. Complications associated with a thin endometrium can be addressed [4]. By looking into the molecular pathways and cell reactions generated through PRP injection, more knowledge of its impact on endometrial health is gained. The review article seeks to provide an entire review of the function of PRP in ART, concentrating mostly on its effectiveness and action in treating thin endometrium. By consolidating statistics from systematic critiques, randomized managed trials, and narrative critiques, this review aims to offer a complete viewpoint on the capability of PRP as a recuperation intervention for enhancing endometrial receptivity and reproductive effects. Through an entire evaluation of the relevant literature and clinical studies, this assessment contributes to the developing panorama of reproductive treatment by clarifying the benefits and problems related to incorporating PRP into endometrial remedy regimens.

The significance of PRP in ART

The use of PRP has been refined in many scientific fields, especially in ART [5]. The efficacy of PRP in improving live births and medical pregnancy outcomes among ladies experiencing recurrent implantation failure (RIF) at some stage of in vitro fertilization (IVF) has been shown through scientific research [6]. Nevertheless, the available facts concerning the efficacy of PRP in ART are limited and of subpar satisfaction [7]. In conjunction with ART, PRP has also been examined for its potential use healing in vitiligo and tendinopathies [8,9]. The use of PRP in ART represents a vast change within the area, marking a new era in regenerative medicine in the realm of fertility. The complicated interaction among growth factors and bioactive proteins in PRP highlights its crucial significance in improving reproductive results. Although the first findings were disappointing, it is far crucial to establish standardized techniques for preparing PRP if assurance of consistency and dependability in future research is to be attained. This highlights the experimental character of PRP usage until dependable large-scale randomized controlled trials are possible [4]. The immunomodulatory actions of PRP play a critical function in shaping an appropriate milieu for embryo implantation, lowering infection, and stimulating tissue repair in the reproductive canal. This comprehensive action not only tackles RIF but also has promise in resolving unexplained infertility, giving a custom-designed therapy strategy adapted to male or female patient requirements. The synergistic ability of PRP in conjunction with current ART strategies emphasizes its adaptability as a supplementary intervention, boosting treatment effectiveness and opening the door for individualized methods in enhancing reproductive effects [6]. The nonuniformity in PRP preparation is a challenge, probably influencing the findings and reproducibility of studies in reproductive medicine. The extraordinary strategies of PRP development, extending from whole blood series to commercially available kits, underline the need for an agreement on preparation approaches to guarantee consistency [10].

The use of PRP has been extensively investigated in many medical contexts, notably within the realm of ART [5]. The efficacy of PRP in enhancing live birth and clinical pregnancy rates among women experiencing RIF during IVF has been shown via clinical studies [6]. Nevertheless, the available data regarding the efficacy of PRP in ART is restricted and of substandard quality [7]. In conjunction with ART, PRP has also been examined for its potential therapeutic use in vitiligo and tendinopathies [8,9].

**Figure 1:**
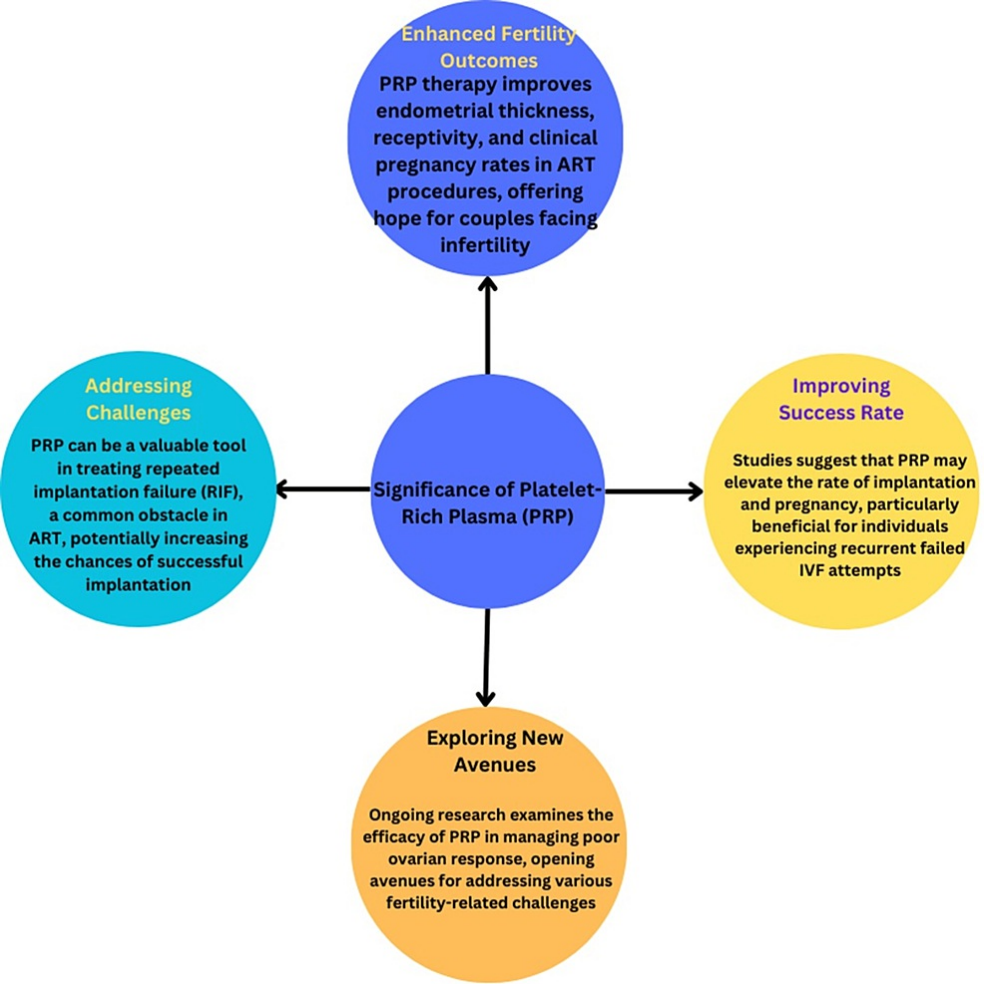
The significance of PRP in ART PRP: Platelet-rich plasma; ART: assisted reproductive technology This figure is created by Priti Karadbhajane and Hellen Yayra Dzoagbe

The use of PRP in ART represents a significant change in the field, marking the advent of a new era in regenerative medicine within the realm of fertility. The complex interaction between growth factors and bioactive proteins within PRP highlights its crucial role in enhancing reproductive results. Although the first findings were disappointing, it is crucial to establish standardized techniques for preparing PRP to guarantee consistency and dependability in future research. This highlights the experimental character of PRP usage until reliable large-scale randomized controlled trials are accessible [4]. The immunomodulatory actions of PRP play a vital role in shaping a suitable milieu for embryo implantation, reducing inflammation, and stimulating tissue repair inside the reproductive canal. This comprehensive action not only tackles RIF but also has promise in resolving unexplained infertility, giving a customized therapy strategy adapted to individual patient requirements. The synergistic potential of PRP in combination with current ART procedures emphasizes its adaptability as a supplementary intervention, boosting treatment effectiveness and opening the door for individualized tactics in improving reproductive outcomes [6]. The lack of uniformity in PRP preparation procedures offers a substantial difficulty, possibly influencing the findings and reproducibility of research in reproductive medicine. The different techniques of PRP preparation, extending from whole blood collection to commercially accessible kits, underline the need for an agreement on preparation procedures to guarantee consistency and dependability in study outputs. The developing environment of PRP in ART necessitates a coordinated push toward standardization to harness its full potential in improving fertility treatments [10]. Figure 1 shows the diagrammatic representation of the significance of PRP in ART. 

PRP has shown encouraging results in the treatment of solid vitiligo lesions, and significant improvements have been observed compared to control groups. For tendinopathy, the use of PRP remains controversial due to inconsistencies in preparation procedures. Overall, although PRP holds promise for ART and other medical interventions, more research is needed to prove its utility and implement it successfully.

## Review

Endometrial receptivity

Endometrial receptivity is an essential element for wholesome pregnancy. It refers back to the time in which the endometrial epithelium became prone to blastocyst implantation. This window of implantation happens at some stage in days 20-24 of the menstrual cycle in women [11]. Despite enhancements in information regarding endometrial physiology, the formation of endometrial receptivity remains a biological mystery [12]. Endometrial receptivity is a tremendous part of the implantation in ART because it represents the capacity of the uterine lining to be in a perfect situation for embryo attachment and growth, sooner or later leading to pregnancy. This system synchronizes embryo growth and endometrial preparation, which is vital for implantation success. The window of implantation, often between days 20 and 24 of a 28-day cycle, is when the endometrium is most liable to embryo implantation [13]. Researchers have discovered many elements that contribute to endometrial receptivity, which includes hormone control and molecular approaches [14]. However, powerful biomarkers for measuring endometrial receptivity are missing [15]. Insufficient receptivity of the endometrium and abnormal embryo-endometrial interaction describes in detail roughly two-thirds of human infertility. Current techniques for detecting endometrial receptivity, together with the thickness of the endometrium and the endometrial receptivity assay, do not reliably enhance scientific outcomes measured by way of several live births [16].

Diagnostic strategies primarily based on molecular markers have been developed; however, their everyday scientific use is not supported by using existing information. Gynecologic disorders like polyps, fibroids, adenomyosis, and continual endometritis may severely influence endometrial receptivity and the effectiveness of assisted reproductive technologies. Personalized embryo transfer, based on the individual's receptive transcriptome profile and endometrial microbiota balance, is an approach to enhance pregnancy outcomes. Further study is required to understand endometrial physiology better and develop molecular tests for directing clinical therapy.

Role of Endometrium in IVF Success

Many variables associated with the embryo affect the effectiveness of IVF. Intestinal microbial dysfunction and changes in the implantation window can lead to RIF [17]. Ultrasound for fetal characteristics such as blood flow, echo pattern, and size can be correlated with the IVF pregnancy rate [18]. Endometriosis, a gynecological illness, does not dramatically impair folliculogenesis and embryo development in IVF, even though it is connected with an extra hazard of sudden adverse reaction [19]. In endometriosis, the implantation competency of splendid euploid blastocysts is not always confined, indicating a similar pregnancy result in comparison to people without endometriosis [20]. Endometrial length (EL) of ≥39 mm has been mounted as an excellent cutoff factor for IVF/intracytoplasmic sperm injection (ICSI) pregnancy rates, demonstrating the relevance of endometrial receptiveness. According to a recent meta-analysis, the subsequent characteristics confirmed better predictive values for successful treatment: a trilaminar sample, endometrial thickness of >7 mm, and endometrial volume of >2 mL [13]. Understanding the molecular pathways influencing endometrial receptivity is essential for enhancing IVF success. Hormonal modulation, molecular signaling pathways, and extracellular matrix (ECM) remodeling are implicated in endometrial receptivity, with inadequate levels of estrogen and progesterone proven to result in implantation failure and infertility [12]. The endometrium's characteristics in IVF effectiveness are multifaceted, comprising hormone management, molecular techniques, and endometrial receptivity assessment. Understanding those characteristics is essential for optimizing IVF effects and improving the percentages of a successful pregnancy.

Impact of PRP on Endometrial Receptivity

Implantation of PRP intrauterine has shown promise in improving endometrial receptivity and thickness in cases where thin endometrial lining (EMT) and RIF are found in infertile ladies [21,22]. PRP consists of growth elements like transforming growth factor (TGF), vascular endothelial growth factor (VEGF), insulin-like growth factor (IGF), platelet-derived growth factor (PDGF), and epidermal growth factor (EGF), which are implicated in sub-endometrial angiogenesis and endometrial receptivity [23]. PRP has emerged as a viable treatment technique in reproductive medicine, substantially improving endometrial receptivity. The sensitive interaction between increased elements and cytokines in PRP has been established to increase cell proliferation, angiogenesis, and tissue regeneration in the endometrium. This recovery functionality of PRP bears vital implications for enhancing implantation rates and, sooner or later, improving the efficacy of ART with IVF. Studies have clarified the techniques through which PRP impacts endometrial receptivity. By growing the recruitment and activation of endometrial stem cells, PRP improves the restoration of injured endometrial tissue and increases the release of crucial additives in embryo implantation.

Furthermore, PRP's anti-inflammatory characteristics are crucial in generating a favorable milieu in the endometrium, conducive to embryo implantation and subsequently establishing a pregnancy. Preclinical trials found better endometrial proliferation rates, expression of proliferative genes, and pregnancy rates with PRP infusion [24]. In vitro studies have shown improved stromal and mesenchymal cell proliferation, expression of regenerative enzymes, and augmentation in cell migration using PRP.

Techniques for PRP Preparation

**Figure 2:**
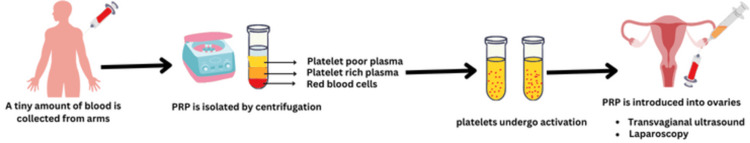
Schematic representation of the preparation of PRP PRP: Platelet-rich plasma Figure created by Hellen Yayra Dzoagbe and Priti Karadbhajane

PRP can be manufactured using numerous procedures. One frequent method is the unmarried-centrifugation procedure, wherein the blood is centrifuged as soon as possible to isolate the PRP from other components. Another approach is the double-centrifugation procedure, which accommodates two spins to concentrate the platelets further. Studies have verified that the double-centrifugation method leads to multiplied platelet amount and yield compared to the single-centrifugation method [25]. In a research by Jo et al., the impact of centrifugation length and gravitational force (g) on the ratio of platelet recovery in PRP was evaluated [26]. They discovered that a higher centrifugation force (g) connected to a higher platelet recovery ratio. For example, presenting an acceleration of 900 × g for five minutes in the first spin stage yielded a superior efficiency of about 92%. In some other research, 9 ml of complete blood was processed, and the platelet amount measured 310.7 ± 78.5 × 103/mm^3 ^after the first spin step. The second spin degree is also critical for concentrating the platelets. Different centrifugation strengths and durations may be used in this stage to produce the specified platelet concentration. For example, a 1500 × g centrifugation for 15 minutes yielded a maximum efficiency of about 84% and a platelet concentration of 633.2 ± 91.6 × 103/mm^3^ [27-30], Schematic instance of PRP education (Figure 2).

The procedures used in manufacturing PRP consist of single and double centrifugation, each with advantages and downsides. Singular centrifugation is a direct and efficient technique of removing red blood cells from the blood by distinguishing blood components with a single spin. However, since it concentrates platelets within a particular density variety, it decreases platelet content. It is found in the entire blood and may not be as powerful as the whole blood. Conversely, the two-spin double-centrifugation technique yields a better platelet concentration and promises better patient results. Notwithstanding its benefits, this approach is extra demanding and requires specialized gear and understanding [31,32]. An essential element in evaluating the effectiveness of PRP preparation is platelets' awareness, yield, and performance. It has been shown that double-spin strategies produce significantly larger platelet concentrations, increasing PRP's ability to be used in healing. They ensure an extra effective PRP product for remedy by imparting a more efficient platelet preparation. The double-spin method is a desired choice for optimizing the therapeutic effects of PRP in medicine because of its effectiveness in concentrating platelets, which improves outcomes in regenerative remedies. Research papers and clinical studies provide insightful records on preparing PRP. Diverse PRP formulations have been added to clinical practice due to the authors' definition of PRP, which has enhanced portions of red blood cells, various leukocytes, fibrin, and bioactive proteins. Precise preparation methods are crucial because uneven patient outcomes have been reported due to nonstandardized product preparation [33]. 

The turn-down turn-up and syringe-only techniques are novel processes recommended to streamline PRP practice [33]. With the syringe-only approach, a closed technique where the blood product remains inside a widely convenient clinical equipment, a 5 ml syringe is rectified for centrifugation. The sterility and appropriateness of the blood product for intravenous injections are assured through this method. Furthermore, the turn-down turn-up technique improves the efficiency of PRP production by using a single 10 mL syringe and two vacutainers for a double-spin system [33].

Selecting between closed and open procedures is a crucial issue of PRP production. While the closed technique uses industrial technology for production, the open method involves the open production of PRP. To lessen the possibility of contamination, glass vacutainers are endorsed over plastic ones. Aseptic measures are critical to preventing infections, all through PRP preparation. It is crucial to realize the basics of centrifugation while making PRP to guarantee reliable and efficient outcomes. Therefore, to maximize the therapeutic results of PRP in several clinical applications, it is crucial to pick out the proper PRP production methods based on variables that include platelet concentration, yield, and efficiency. Utilizing advancements in PRP preparation techniques underscores the importance of precision, uniformity, and adherence to established protocols, potentially boosting the efficacy of PRP treatments.

Mechanism of action

How PRP Affects Endometrial Healing and Regeneration

Through numerous approaches, PRP promotes endometrial regeneration and restoration. Improved endometrial regeneration proceeds from PRP therapy because it upregulates the pathways for retinol metabolism and ECM receptor interplay [34]. In PRP-prompted endometrial regeneration, melanotransferrin (MELTF) is a crucially upregulated gene [35]. PRP stimulates the expression of genes related to mobile proliferation, tissue regeneration, proinflammatory response, and antimicrobial outcomes in undifferentiated human endometrial stromal cells (HESCs). By blockage of phosphoinositide 3-kinase signaling, PRP reduces the expression of genes connected to infection and mobile proliferation in decidualized HESCs. PRP may also accentuate immunological tolerance during the secretory stage and thicken the endometrium at some stage in the proliferative segment. PRP commonly improves tissue regeneration and reproductive consequences by influencing many pathways and gene expressions that guide endometrial repair and regeneration. PRP impacts endometrial repair and regeneration via a complex process encompassing both molecular and cell mechanisms.

Activation of Platelets in PRP

PRP is made from a vast quantity of activated platelets subjected to centrifugation or different approaches that cause the platelets' inner growth elements and cytokines to be released. The release of bioactive proteins called growth factors in PRP is dependent on the activation of platelets, which is a crucial first step. This activation starts after centrifugation and stimulates platelet degranulation, leading to the discharge of growth factors that play essential roles in mobile mitosis, angiogenesis, chondrogenesis, and chemotaxis. Launching these growth elements is crucial when you consider that they cause the restoration cascade in injured tissues, leading to the therapeutic effects of PRP [36]. PRP can be activated by using some techniques, which include the usage of thrombin, fibrin, or calcium chloride. It has been shown that activating platelets before therapy will increase the release of the correct growth elements, growing PRP's therapeutic effectiveness. Studies have indicated that activated PRP demonstrates progressed outcomes compared to nonactivated PRP, necessitating the activation PRP for maximum advantages in therapy [37]. One vital stage of PRP is the activation of platelets, which sets off the discharge of growth elements essential for tissue regeneration and restoration. Efficient activation techniques, such as thrombin, fibrin, or calcium chloride, are critical for optimizing platelet-derived prosthetic platelet therapy in several clinical contexts.

Release of Growth Factors

Platelets that have been activated secrete a range of growth elements that are critical for tissue regeneration and restoration, such as VEGF, PDGF, and transforming growth factor-beta (TGF-β) [3]. The promotion of angiogenesis, or the introduction of new blood vessels, is a famous function of VEGF. Angiogenesis is essential for offering vitamins and oxygen to tissues for the healing duration. PDGF is critical for cell migration, proliferation, and synthesizing ECM elements required for tissue healing. The multifunctional TGF-β controls some cellular features, including immune response regulation, cellular proliferation, and differentiation.

The mixed impact of those growth elements, generated through activated platelets, quickens tissue recuperation, increases cell division, and fosters tissue regeneration. Comprehending the strategies involved in releasing those growth factors is essential for optimizing the reparative capability of PRP for various medical uses in ophthalmology, orthopedics, wound recovery, and other healing measures [38].

Stimulation of Angiogenesis

Angiogenesis is triggered by the growth elements produced by activated platelets. This system is essential to provide oxygen and nutrients to the endometrial tissue, fostering repair and regeneration [39]. The growth factors generated with the aid of activated platelets play an essential part in stimulating angiogenesis and improving the new blood vessels. These growth elements are essential in encouraging the formation of new blood vessels, which is vital to providing oxygen and vitamins to tissues, including the endometrium, to facilitate procedures of restoration and regeneration [40,41].

Numerous proangiogenic materials, such as essential fibroblast growth factor (bFGF), VEGF, and different angiogenesis stimulators, are launched via platelets. These molecules each start and manipulate the angiogenic cascade. A balanced reaction is ensured by the segregation of pro- and antiangiogenic proteins within platelets, which allows for the regulated encouragement of angiogenesis even while restricting immoderate vessel formation or aberrant vascularization [42]. It is critical to realize the mechanisms via which platelets promote angiogenesis and manage the ratio of pro- to antiangiogenic materials in several physiological processes, which include tissue repair, tumor development, and wound restoration. Activated platelets are critical in regulating angiogenesis, as proven through their coordinated launch of growth elements. This highlights the significance of platelets in promoting tissue regeneration and repair through the production of new blood vessels [43].

Cell Proliferation and Collagen Synthesis

Growth factor-activated signaling pathways boost the variety of cells collaborating in tissue recovery by enhancing cellular proliferation. They additionally encourage the creation of collagen, which is vital for giving the endometrial tissue structural support. One essential growth element that is recognized to promote collagen production and connective tissue growth factor (CTGF), which can be vital for tissue restoration, is TGF-β1. By inducing CTGF, TGF-β1 triggers Smad2/3 signaling pathways, which causes collagen formation. Moreover, TGF-β1 and CTGF each promote the expression of heat surprise protein 47 (HSP47), critical for collagen synthesis, illustrating the complicated pathways by which growth factors manage collagen formation and tissue restoration [44]. Growth elements have a critical function in facilitating tissue regeneration and restoration due to the fact that they stimulate mobile proliferation and collagen formation. To fully use those growth elements' healing capacity in several clinical settings, which includes wound restoration, fibrosis, and tissue repair inside the endometrium and other organs, it is vital to realize how those elements improve cell activities and collagen synthesis [45].

Recruitment of Stem Cells

PRP facilitates draw-in and prompt endometrial stem cells, which may develop into distinct types of cells concerned with tissue regeneration and useful resources in the restoration system. PRP's ability to stimulate and appeal to endometrial stem cells underscores its regenerative capacity in facilitating the body's inherent restoration approaches. PRP promotes the differentiation of those stem cells into certain cells, which tends to aid in tissue restoration, collagen formation, and overall tissue recovery. This procedure highlights the healing advantages of PRP in encouraging scar restoration and helping with the repair of injured endometrial tissues, presenting a viable method for boosting tissue regeneration and repair. PRP remedy has been proven to be influential in promoting tissue regeneration and facilitating the recovery system because of its ability to draw and activate endometrial stem cells. Through PRP therapy, healthcare experts may further augment the body's innate healing techniques, inspire tissue restoration, and facilitate the regeneration of impaired endometrial tissues [38].

*Enhanced ECM* *Deposition*

PRP growth elements encourage the collagen and fibronectin of the ECM to be deposited, which is vital for the endometrial tissue to be rebuilt and restructured. These growth elements enhance the synthesis of fibronectin and collagen, critical aspects of the ECM, that provide tissues with structural integrity and support. The growth factors allow the deposition of collagen and fibronectin, which might be necessary for the remodeling and regeneration of endometrial tissue and the protection of its structural integrity [46].

The ECM is critical for assisting tissue recuperation procedures, controlling activities of the cell, and providing cellular scaffold. PRP's growth factors enhance the ECM's collagen and fibronectin deposition, which promotes the structural framework required for cellular migration, proliferation, and differentiation in the direction of tissue regeneration. Rebuilding injured endometrial tissue and ensuring appropriate reorganization is essential for encouraging the best possible recuperation effects [44]. All matters considered, PRP's boom factors' functionality to promote collagen and fibronectin deposition in the ECM highlights how essential they are for aiding in the regeneration of endometrial tissue. PRP creates a favorable environment for tissue restoration, transformation, and purposeful recovery in the endometrium by encouraging the reconfiguration of the ECM via accelerated collagen and fibronectin deposition. This method demonstrates PRP's potential for regeneration in aiding scar-free recovery and encouraging most viable tissue regeneration inside the endometrium. The growth factors in PDGF, TGF-β, and different PRP-derived substances encourage the expression of collagen and fibronectin. The mechanisms that bring about superior fibronectin and collagen synthesis and ECM incorporation are initiated when those growth factors bind to receptors on cells which include fibroblasts and keratinocytes. Fibronectin is a "master organizer" inside the matrix assembly method, promoting the adhesion, migration, proliferation, and contraction of cells, all critical steps in the recuperation of wounds [47].

The deposition of collagen and fibronectin, which is facilitated by way of PRP growth elements, is vital for the reconstruction of injured tissue with regard to endometrial tissue. Collagen guarantees appropriate tissue structure and characteristics by giving the uterine lining structural balance. The restoration technique includes fibronectin in cell adhesion and migration, which is critical for the regeneration of healthy endometrial tissue with minimum scarring. Tissue regeneration is made feasible via the coordinated activity of PRP growth elements, which encourage the deposition of collagen and fibronectin within the ECM. Through the augmentation of these essential components, PRP allows the transformation of endometrial tissue, encouraging restoration without scarring and ensuring the survival and functioning of the regenerated tissue [48].

Creation of Pro-regenerative Microenvironment

To reduce damage caused by inflammation and to promote healing, PRP produces circumstances which are favorable for tissue repair by moderating the inflammatory response and fostering a pro-regenerative milieu.

Optimizing tissue restoration effects calls for PRP's capability to modulate the inflammatory reaction and foster a pro-regenerative milieu. PRP remedy improves the body's natural healing pathways and hastens the repair of injured tissues by lowering irritation and fostering an environment that encourages tissue regeneration. By maximizing endogenous regenerative mechanisms instead of depending on extracellular transplantation, this method seeks to promote purposeful tissue regeneration and offers a feasible method for improving tissue restoration and regeneration in vivo [49].

Resolution of Inflammation

Because PRP has anti-inflammatory traits, it may lessen inflammation inside the endometrial tissue, which lowers fibrosis (the introduction of excessive scar tissue) and improves the surroundings in which regeneration can occur. PRP's ability to reduce endometrial tissue irritation is essential in preventing fibrosis, which may additionally obstruct healthy tissue regeneration and repair. Through the management of irritation and its deleterious consequences, PRP treatment seeks to limit fibrosis and foster a pro-regenerative milieu to be able to facilitate premier tissue repair. PRP's ability to reduce inflammation highlights how it could aid in restoration and accentuate the outcomes of endometrial tissue regeneration. Therefore, PRP's anti-inflammatory properties are essential for lowering inflammation in endometrial tissue, which lowers fibrosis and fosters regeneration. PRP treatment addresses inflammation-related issues and supports the recovery effects inside the endometrium by way of controlling the inflammatory response, providing a feasible strategy for encouraging tissue repair and regeneration [50].

Optimization of Endometrial Receptivity

PRP subsequently improves endometrial receptivity for embryo implantation, increasing the probability of favorable pregnancy outcomes through its complicated influence on angiogenesis, cellular proliferation, stem cellular recruitment, ECM deposition, and inflammation resolution. Through several pathways, PRP has been shown to improve endometrial receptivity for embryo implantation. Because PRP has high content of growth elements and cytokines, it promotes vascularization, cell proliferation, and anti-inflammatory outcomes, and reduces fibrosis, all of which improves endometrial receptivity. As PRP produces growth factors inclusive of VEGF, PDGF, EGF, TGF, and IGF1, which promote endometrial cellular proliferation and regeneration, research has shown that PRP is essential for cell proliferation, regeneration, and differentiation. PRP has additionally been proven to promote endometrial receptivity by upregulating adhesion molecule expression, drawing in stem cells, and promoting endometrial cell migration. To add to this, PRP has been connected to multiplying levels of Hoxa10, an important endometrial receptivity marker, suggesting that it has a beneficial effect on endometrial preparation for embryo implantation. Even with those encouraging outcomes, additional investigation and clarification are still needed to decide the best time for applying PRP and the dose needed for maximizing endometrial responsiveness [4].

Clinical studies and efficacy

The effectiveness of PRP therapy has been investigated in some therapeutic contexts. PRP has been researched as a possible remedy option in the context of ART for ladies with recurrent ART failure. Anitua et al. recommend that PRP treatments can be beneficial in patients who do not positively respond to standard ART [51]. However, further research is required to validate this recommendation [46]. Table 1 shows the research related PRP done by other researchers and its characteristics.

**Table 1:** Principal characteristics of the chosen research RIF: Repeated implantation failure; RPL: recurrent pregnancy loss; HRT: hormone replacement therapy; ET: endometrium; NR: not reported Table was created by Priti Karadbhajane and Hellen Yayra Dzoagbe

Study	Cause of failed embryo transfer	PRP Obtention Protocol	Platelet concentration	Application method
Allahveisi et al. (2020) [52]	RIF	1700 g 12 min; 3300 g 7 min	411 × 103–1067 × 103/uL	Intrauterine infusion
Farimani et al. (2021) [53]	RIF (≥3)	1200 rpm 12 min; 3300 rpm 7 min	4–5 times	Intrauterine infusion
Zargar et al. (2021) [54]	RIF (≥2)	12,000 g 10 min; 12,000 g 10 min	NR	Intrauterine infusion
Nazari et al. (2022) [55]	RPL	1200 rpm 12 min; 3300 rpm 7 min	4–5 times	Intrauterine infusion under ultrasound guidance
Eftekhar et al. (2018) [30]	Poor endometrial response (ET < 7 mm) to HRT	1600 g 10 min; 3500 g 5 min	4–5 times, 2000 lymphocyte	Intrauterine infusion

Comparison of Pregnancy Rates With and Without PRP in ART

PRP in ART has improved pregnancy rates. In patients with RIF, intrauterine perfusion of autologous PRP dramatically enhanced endometrial receptivity, implantation, and pregnancy rates [22].

Similar to this, Stojkovska et al. observed increased implantation and live birth rates in poor ovarian responders (PORs) treated with a transvaginal intraovarian injection of PRP before IVF/ICSI [56]. In PORs receiving IVF/ICSI, the studies sought to evaluate the effectiveness of PRP remedy by using transvaginal intraovarian injection, with a specific emphasis on implantation and live birth rates. The study compared the effects of PRP therapy for PORs, juxtaposed to the control group. It is possible that the studies documented live pregnancy results, implantation rates, and monitoring of patients all through IVF/ICSI cycles. According to the findings of Stojkovska et al.'s research, transvaginal intraovarian PRP injections in PORs receiving IVF/ICSI may additionally increase the probability of a success in implantations and live births [56]. This research highlights the function of PRP as a likely technique to improve reproductive effects in difficult cases and proffers a therapy for patients with poor ovarian response [56].

To better apprehend how intrauterine PRP injection impacts pregnancy rates in those with RIF, Rageh et al. conducted a research using granulocyte colony-stimulating factor (GCSF) and PRP to enhance pregnancy results in such patients, which was the motive of the study [57]. Rageh et al. used a nonrandomized scientific study method to determine how intrauterine PRP affected the results of ICSI in patients with RIF [57]. The outcomes in patients receiving GCSF and PRP have been compared in the studies. The findings confirmed tremendously higher clinical pregnancy rates with PRP than GCSF, indicating that individuals with RIF would gain from this approach. The findings showed that large randomized clinical research is important to establish the effectiveness of PRP in enhancing pregnancy results in patients with RIF, even in the face of attempts to account for confounding variables [48].

To determine if PRP may additionally boost the amount of live births and clinical pregnancy rates among women with RIF undergoing IVF, Dorofeieva and Boichuk completed a comprehensive study [58]. The motive of the systematic review was to evaluate the body of literature on the outcomes of intrauterine PRP infusion throughout pregnancy, with a particular emphasis on live birth and clinical pregnancy rates among RIF ladies undergoing IVF. A thorough search of many databases, like PubMed, Scopus, Cochrane, and Web of Science, was done by Dorofeieva and Boichuk [58]. They used cohort studies and randomized and nonrandomized medical trials that evaluated the use of intrauterine PRP in infertile ladies. The Cochrane Risk of Bias Tool and the Newcastle-Ottawa Scale were examples of good analytical instruments that were used.

The systematic review provided proof that PRP is effective in improving IVF success in patients, particularly those with recessive infertility. Notwithstanding the encouraging results, the need for further large-scale, multicenter, randomized scientific research to establish the optimum PRP doses and pinpoint the ideal groups who would benefit most from this course of remedy was highlighted in the review [59].

**Figure 3:**
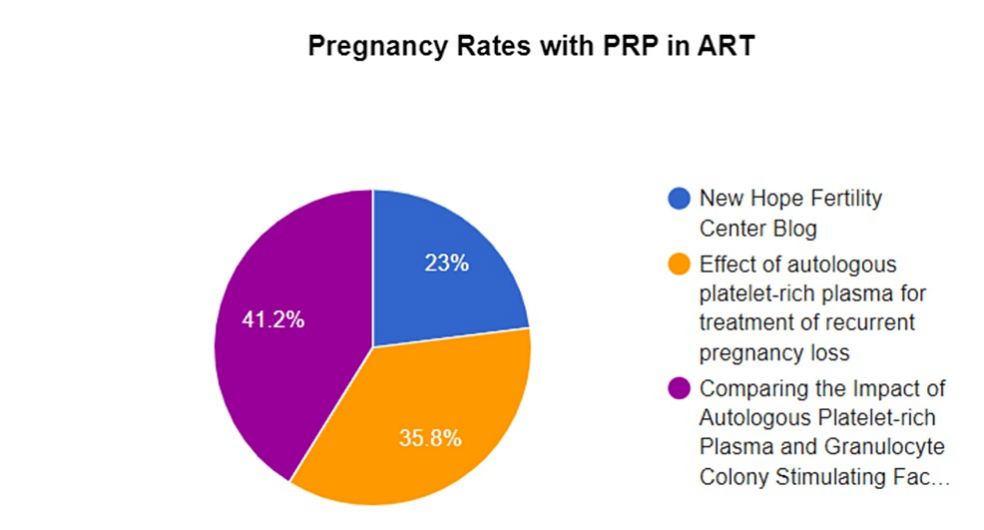
Pregnancy rates with PRP in ART PRP: Platelet-rich plasma; ART: assisted reproductive technology This figure was created by Hellen Yayra Dzoagbe and Priti Karadbhajane

Depending on the type of remedy, patient groups, and study design, these studies show various pregnancy outcomes of PRP in ART, ranging from 15% to 40%. To prove that PRP consistently improves pregnancy outcomes after ART, further studies are required. Figure 3 illustrates pregnancy rates with PRP in ART.

Safety concerns of PRP in ART

Because of its ability to enhance the results of assisted reproduction, PRP has attracted interest in ART. When thinking about using PRP in ART, there are critical safety considerations that should be considered. Because PRP is made from the patient's blood, there are dangers related to managing blood components in the course of PRP preparation, including the possibility of infection and immunogenic responses. Additionally, there are unknowns about the prolonged implications of PRP on the well-being of the mother and the child. A principal concern is also related to regulatory and ethical principles, which highlight the use of particular protocols, strict supervision, and ethical standards to assure patient safety and the effectiveness of PRP therapies in reproductive medicine. To completely examine the safety profile of PRP in ART and remedy these safety issues, further research consisting of full-size randomized managed trials and systematic critiques is needed to maximize its sensible applicability in reproductive medicine.

Risk of Infection

Handling blood elements all through the manufacture of PRP increases the chance of impurities and infection if suitable sterile measures are not applied. Numerous research studies have emphasized how essential it is to keep stringent requirements so one can reduce this chance. For instance, research has shown that PRP has considerable antimicrobial action against microorganisms, which include *S. aureus*, *Neisseria gonorrhoeae*, and group A *Streptococcus*, highlighting its ability as an infection-preventive agent [60]. Furthermore, PRP's antimicrobial traits and potent pathogen-killing abilities have been stated for its use in the treatment of tissue infections, making it a useful adjuvant remedy for infection control. PRP is useful in encouraging recuperation and tissue repair; nonetheless, there may still be a chance of infection; therefore, as a way to guarantee the patient's safety and treatment effectiveness, strict aseptic protocols ought to be followed for the duration of PRP production and transfer [61].

Immunogenic Reactions

Since PRP is made from the patient's blood, immunological reactions may affect how well ART treatments work. A major risk linked with using PRP in ART is immunogenic responses. PRP, made from the patient's blood, may cause immunological reactions that affect how well ART treatments work. According to research, PRP may have immunomodulatory effects that affect the immune system's reaction. Research has shown that patient-specific immune responses vary widely, underscoring the significance of individual immune cell and cytokine profiles in defining PRP composition. This variation in immune profiles might result in different reactions to PRP therapy, which can impact the treatment's effectiveness for ailments like osteoarthritis.

Moreover, the intricacy of immunological interactions within PRP formulations is highlighted by including granulocytes and lymphocytes, which are important modulators of regeneration in PRP. The immunomodulatory characteristics of platelet-derived plasma are shaped by people's distinct adaptive immunological profiles, which are impacted by variables such as age, sex, and physical activity. To reduce the possibility of immunogenic reactions and maximize the safety and efficacy of PRP in ART operations, it is crucial to comprehend the complex interplay of PRP, immunological responses, and patient-specific variables [62].

Effect on Embryo Development

Research indicates that PRP may additionally affect the growth and development of embryos, prompting questions concerning how this could affect the effectiveness of ART treatment alternatives as a whole [21]. The possible effects of PRP on embryo development within the context of ART have been studied. Research has validated that PRP can in fact affect the growth and development of embryos, questioning how this can affect the effectiveness of ART remedy entirely. PRP has been discovered to promote the migration and proliferation of endometrial mesenchymal stem cells, according to research by Wang et al. (2018) that examined the substance's action on those cells [63]. Furthermore, Anitua et al.'s complete evaluation and meta-analysis from 2023 highlighted the functional effectiveness of PRP in enhancing pregnancy results for ladies who had a record of unsuccessful embryo transfers [51]. The particular pathways through which PRP affects embryo development and the lasting ramifications of such effects continue to be subjects that need more exploration, even though modern studies offer insights into the possible advantages of PRP in ART. To ensure the safety and effectiveness of PRP utilization in enhancing reproductive results, it is a must to address the issues and completely understand its usefulness in ART.

PRP Combined With Endometrial Scratching

In the world of ART, the combination of PRP and endometrial scraping shows promise, especially for patients having RIF. This novel remedy combines endometrial scratching to boost endometrial receptivity and enhance outcomes of pregnancy with intrauterine PRP injection having a concentrated supply of growth factors and cytokines from the patient's blood [64]. The mixed advantages of PRP and endometrial scratching in improving endometrial development, receptivity, and embryo implantation justify this method. It has been proven that endometrial scratching, a technique that causes mechanical damage to the endometrium, increases endometrial receptivity and triggers a regenerative response by encouraging the release of cytokines and proteins connected to implantation [65].

Research reveals that patients with RIF receiving ART, such as frozen embryo switch and intrauterine PRP injection, may markedly enhance endometrial thickness, implantation success, and pregnancy rates [6]. PRP's growth factors and cytokines are crucial for improving the division, regeneration, and proliferation of endometrial cells. This improves endometrial receptivity and gets the endometrium geared up for a successful embryo implantation. Furthermore, there can be a synergistic effect between PRP and endometrial scratching because the latter creates surroundings that let PRP act at the endometrium in a regenerative and growth-enhancing manner. PRP's ability to deal with the multifactorial nature of RIF, which results from immunologic factors, aberrant embryo-endometrial reaction, embryonic abnormalities, and poor endometrial receptivity, makes it a potentially therapeutic combination when paired with endometrial scratching [65]. PRP remedy mixed with endometrial scratching is an especially superior reproductive remedy approach that has a big capacity for those who are having trouble with the implantation of their embryos. This modern-day remedy maximizes endometrial receptivity, raises pregnancy rates, and increases the chance of successful embryo implantation through the use of PRP's restorative characteristics. The scientific basis of this method highlights how it can transform fertility remedies by focusing on underlying endometrial diseases and creating an environment that is favorable for a successful pregnancy establishment [63,65].

PRP Combined With Embryo Transfer Techniques

Combining present-day embryo technique with PRP therapy is a unique method in reproductive medicine that aims to improve endometrial receptivity and pregnancy outcomes. Concentrated delivery of growth factors along with TGF-β1 and PDGF works with embryo transfer strategies to improve endometrial thickness, angiogenesis, cell proliferation, and reduce inflammation. These hopeful effects have been observed with PRP [66]. This novel treatment method makes use of PRP to generate a pro-regenerative milieu in the endometrium, promoting tissue regeneration and repair which can be vital for a successful embryo implantation and pregnancy development.

The effectiveness of using PRP along with embryo transfer methods to improve endometrial receptivity and enhance pregnancy rate is supported by medical research, particularly in those with a record of RIF. Research has shown that intrauterine infusion of PRP significantly improves endometrial thickness, which is a critical factor connected to better pregnancy rates in women with less fibrosis [66]. This integrated approach affords a tailor-made and powerful technique for improving reproductive results in people who are having problems with implantation, boosting endometrial development, and setting up a favorable environment for successful embryo implantation. Furthermore, their complex consequences on cell proliferation, vascularization, anti-inflammatory traits, and reduction of fibrosis within the endometrium are proven to aid the mechanistic insights into the synergistic outcomes of PRP and embryo switch approaches. This complete therapy method stimulates tissue repair and regeneration while lowering inflammatory responses which can jeopardize endometrial fitness. It does this by way of the usage of the powerful peptides, growth factors, and cytokines present in platelets and are rich in progesterone. By treating underlying endometrial pathologies and creating surroundings, this is favorable to successful conception and pregnancy, while PRP therapy and embryo switch strategies represent a complicated intervention that can revolutionize fertility treatments [67].

Therapeutic applications of PRP

PRP has been proven as a promising treatment option in some medical specialties, like sports, dermatology, and orthopedics. PRP is used because it stimulates tissue regeneration and repair by liberating growth factors and cytokines that encourage angiogenesis, cellular proliferation, and the healing of inflammation [31]. PRP has been very well investigated within the subject of orthopedics due to its ability to enhance bone recovery, shorten healing periods, and improve tissue regeneration in ailments such as tendons, ligaments, and cartilage damage. PRP has been proven in numerous trials to be effective in lowering infection and encouraging tissue regeneration in tendons, ligaments, and joints [68]. PRP has been utilized in dermatology to treat a variety of pores and skin-related conditions such as vitiligo, lichen sclerosis, psoriasis, Bechet illness, morphea, androgenetic alopecia, alopecia areata, melasma, and vitiligo. Promising outcomes have been seen in the usage of PRP in boosting pore and skin firmness, decreasing wrinkles, and enhancing pore and skin texture. It has additionally been utilized in sports medicine to improve tissue regeneration, lower inflammation, and hasten bone recuperation in musculoskeletal injuries. PRP has shown promise in enhancing sports performance and decreasing the chances of injury in sports medicine [69]. PRP helps in stimulating cell proliferation, angiogenesis, and inflammation resolution through the production of growth factors consisting of VEGF, TGF-β, and PDGF. Together, those growth factors assist tissue regeneration and restoration in several therapeutic settings. To sum up, PRP has shown noteworthy promise in diverse domains inclusive of orthopedics, dermatology, and sports medicine. Because PRP may additionally promote tissue regeneration and repair with the aid of releasing growth factors and cytokines, it is doubtlessly a powerful option for the treatment of varying clinical diseases [70].

## Conclusions

In ART, PRPs offer a promising alternative, especially when it comes to endometrial complications that accompany fertility treatments. It remains a useful adjuvant for incorporation into therapies, although there is a need for standardization with preparative strategies and further large-scale studies to demonstrate effectiveness in different patient groups. One obvious approach to developing successful, individualized treatments that can address problems such as endometrial thinning, RIF, and other uterine pathologies is uterine absorption, which combines the regenerative and growth-promoting properties of PRP in ART regimens that may pave the way for targeted strategies to enhance endometrial receptivity and health in the future.
